# Posterior α EEG Dynamics Dissociate Current from Future Goals in Working Memory-Guided Visual Search

**DOI:** 10.1523/JNEUROSCI.2945-16.2016

**Published:** 2017-02-08

**Authors:** Ingmar E.J. de Vries, Joram van Driel, Christian N.L. Olivers

**Affiliations:** Institute of Brain and Behavior Amsterdam and Department of Experimental and Applied Psychology, Faculty of Behavioural and Movement Sciences, Vrije Universiteit Amsterdam, 1081BT Amsterdam, The Netherlands

**Keywords:** EEG, priority, selective attention, states, visual search template, visual working memory

## Abstract

Current models of visual search assume that search is guided by an active visual working memory representation of what we are currently looking for. This attentional template for currently relevant stimuli can be dissociated from accessory memory representations that are only needed prospectively, for a future task, and that should be prevented from guiding current attention. However, it remains unclear what electrophysiological mechanisms dissociate currently relevant (serving upcoming selection) from prospectively relevant memories (serving future selection). We measured EEG of 20 human subjects while they performed two consecutive visual search tasks. Before the search tasks, a cue instructed observers which item to look for first (current template) and which second (prospective template). During the delay leading up to the first search display, we found clear suppression of α band (8–14 Hz) activity in regions contralateral to remembered items, comprising both local power and interregional phase synchronization within a posterior parietal network. Importantly, these lateralization effects were stronger when the memory item was currently relevant (i.e., for the first search) compared with when it was prospectively relevant (i.e., for the second search), consistent with current templates being prioritized over future templates. In contrast, event-related potential analysis revealed that the contralateral delay activity was similar for all conditions, suggesting no difference in storage. Together, these findings support the idea that posterior α oscillations represent a state of increased processing or excitability in task-relevant cortical regions, and reflect enhanced cortical prioritization of memory representations that serve as a current selection filter.

**SIGNIFICANCE STATEMENT** Our days are filled with looking for relevant objects while ignoring irrelevant visual information. Such visual search activity is thought to be driven by current goals activated in working memory. However, working memory not only serves current goals, but also future goals, with differential impact upon visual selection. Little is known about how the brain differentiates between current and future goals. Here we show, for the first time, that modulations of brain oscillations in the EEG α frequency band in posterior cortex can dissociate current from future search goals in working memory. Moreover, the dynamics of these oscillations uncover how we flexibly switch focus between memory representations. Together, we reveal how the brain assigns priority for selection.

## Introduction

Trying to find relevant visual information is ubiquitous in everyday life. Such search behavior requires a goal-based representation of what one is looking for, often referred to as the attentional template (Duncan and Humphreys, 1989; Desimone and Duncan, 1995), and which current models assume to be activated in visual working memory (VWM) (Bundesen, 1990; Wolfe, 1994; Itti and Koch, 2001). However, not every VWM representation biases attention evenly (Downing and Dodds, 2004; Houtkamp and Roelfsema, 2006; Carlisle and Woodman, 2011; Olivers and Eimer, 2011). Nor should they: Working memory not only serves our current goals but also allows us to plan ahead and maintain future goals across a series of tasks. To prevent interference, such prospective representations should be shielded from the current task, leading to a proposed distinction in the moment-by-moment task relevance of items held in VWM (Olivers et al., 2011; Oberauer and Hein, 2012; Larocque et al., 2014). On the one hand, currently relevant representations interact with sensory input and/or response output, whereas prospective (also referred to as “accessory”) representations are stored in a more latent, deprioritized state, until later use.

We investigated which electrophysiological measures distinguish between current and prospective relevance of VWM representations for visual search. One difference may be storage. It has been suggested that, whereas currently relevant items are actively maintained, prospectively relevant items are stored passively, through synaptic weight changes (Olivers et al., 2011; Stokes, 2015). Indeed, studies found that multivariate decoding accuracy drops to zero for prospective representations while participants first perform another task (Lewis-Peacock et al., 2012; LaRocque et al., 2013). Where those studies used tasks from rather different cognitive domains (vision and language), we focused on prioritization for visual search. As a measure of storage, we adopted the contralateral delay activity (CDA), a sustained event-related potential (ERP) component known to increase with VWM load (Vogel and Machizawa, 2004). Although the CDA is spatially lateralized, it emerges when feature content is maintained. For example, and important here, the CDA emerges during maintenance of a feature template for visual search (Carlisle et al., 2011; Woodman and Arita, 2011). However, it remains unclear whether the CDA reflects activity that is specific to current relevance or reflects VWM storage in general (Gunseli et al., 2014). We therefore directly compared the CDA for current and prospective relevance in VWM.

Another key characteristic of brain activity during VWM is the modulation of the power and phase of posterior α oscillations (Klimesch et al., 2007; Palva and Palva, 2007). α-band activity is thought to orchestrate visual attention through inhibition of task-irrelevant representations, thereby facilitating activation of task-relevant representations (Jensen and Mazaheri, 2010; Mazaheri et al., 2014; Zumer et al., 2014). Indeed, hemisphere-specific α power over posterior electrodes correlates negatively with attention to the contralateral hemifield (Sauseng et al., 2005b; Kelly et al., 2006; Rihs et al., 2007). Because of the close relationship between attention and VWM (Kiyonaga and Egner, 2013), we hypothesized that posterior α dynamics also reflect the prioritization of different VWM representations for search. Moreover, we hypothesized that such top-down, goal-driven control over memory representations would be supported by changes in functional connectivity, following evidence that long-range α phase synchronization between frontal, parietal, and occipital areas underlies modulation of local oscillations in sensory regions during maintenance (Sauseng et al., 2005a; Palva and Palva, 2011).

We measured EEG while participants performed two consecutive visual search tasks. During the delay before the first search, observers remembered two items: a current template, required for the immediate search task, and a prospective template, used for the second search task, allowing us to dissociate current from prospective relevance. We predicted that hemisphere-specific modulations in α power and interregional phase synchronization should reflect which item has current priority for search. On the other hand, the CDA, if it indeed only reflects storage, should not dissociate between these two different states, unless prospective representations are not stored through sustained activity at all.

## Materials and Methods

### 

#### 

##### Subjects.

Twenty healthy volunteers (ages 23–33 years, 8 female) participated in the experiment for monetary compensation. One participant was replaced due to excessive noise in the EEG recordings. The procedures used were conducted in accordance with the Declaration of Helsinki and were positively reviewed by the faculty's Scientific and Ethical Review Board. Written informed consent was obtained.

##### Task design.

**Figure 1.**
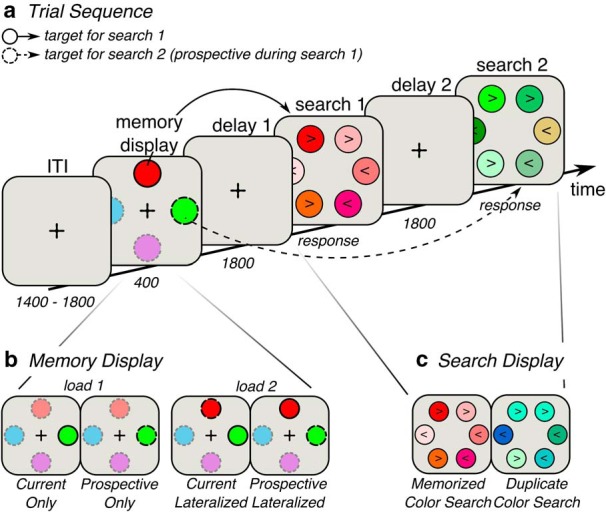
Task design. ***a***, Trial sequence. Participants were presented with two consecutive search displays. Before the search displays, participants were given a memory display indicating which targets to look for in the subsequent search displays. ***b***, There were four types of memory display. In Load 1 conditions, only one target had to be remembered, and it was either designated as the target for the first search (current only; indicated by a solid outline) or for the second search (and thus was prospective during the first search; indicated by a dashed outline). The relevant item was always lateralized. In this case, the remaining search display did not contain a predefined target, and no working memory was required. Instead, in those displays, observers searched for any duplicate color, as illustrated in ***c***. In Load 2 conditions, observers always memorized two items: one for the first search (currently relevant item; solid outline) and one for the second (prospectively relevant item; dashed outline). Either the currently relevant item was lateralized or the prospectively relevant item was lateralized, whereas the other item was presented on the meridian. ITI, Intertrial interval. Object sizes and colors differ from the real experiment, and the opacity for the irrelevant colors in the memory display is set at 50% for illustrative purposes.

On each trial, participants performed two consecutive VWM-guided visual search tasks (Fig. 1*a*). A trial started with a fixation cross on a gray background for a randomly jittered duration of 1400–1800 ms. Next, a memory display with four colored circles surrounding a fixation cross (left, right, top, and bottom) was presented for 400 ms. Depending on the condition, one or two of these colors served as cues as to which color to look for in the subsequent search tasks. This was indicated by the nature of their outlines: the color cue with a full outline indicated the target for the first search display, and thus served as the template for that search display, whereas the color cue with the dashed outline indicated the target for the second search display, and thus was the prospective template with regards to the first search display. The other two colors had a dotted outline and were presented merely for sensory balancing purposes; participants were told they could completely ignore these items. The memory display was followed by a delay period of 1800 ms, during which participants focused on a fixation cross. This was followed by two consecutive search displays, which were presented until response. Between the first response and the second search display, there was another delay period of 1800 ms. The task was to find the memorized color relevant for the respective search display and then indicate the direction of an arrowhead that was plotted inside the target item. Participants responded by clicking a button (placed on both armrests) corresponding to the direction of the arrowhead (i.e., the left hand button for the leftwards pointing arrowhead, and the right hand button for the rightwards pointing arrowhead). The second response was immediately followed by the gray fixation cross of the next trial. If participants did not respond within 5 s, they were shown the message: “Too slow! Please respond faster.” Participants were instructed to fixate on the central fixation cross during the intertrial interval, the memory display, and both delay periods. This setup of trials and timings of displays was the same in all conditions.

Our design contained four main conditions (Table 1; Fig. 1*b*), with as most important manipulations whether the current template or the prospective template was presented lateralized, and whether either one or both templates had to be remembered. In Load 2 conditions, both a current and a prospective template had to be remembered, and there were two versions: In the Current Lateralized condition, the current template was presented left or right from central fixation, whereas the prospective template was presented on the central meridian, above or below fixation. In the Prospective Lateralized condition, the prospective template was presented lateralized, whereas the current template was presented on the meridian. In addition, there were two Load 1 conditions. In these conditions, only one color had to be remembered which was always lateralized, and which was either needed for the first search display (Current Only condition), or for the second search display (Prospective Only condition). In Load 1 conditions, one search task was thus driven by the item in VWM, whereas for the other search task no memorized item was required. Instead, this type of search display contained two circles with identical colors; and rather than trying to find a specific color, the task was to find the duplicate color (which could be any color in the set). Participants indicated the direction of the arrowheads in the items carrying the duplicate color (Fig. 1*c*). For example, when the item was relevant for the second search (Prospective Only*),* the first search display did not require a memory for the target, but observers searched for the duplicate color instead. With this design, we manipulated the current or prospective task relevance that the lateralized memory item acquired before the search itself, enabling us to directly relate lateralized EEG patterns to either a currently relevant or prospectively relevant VWM representation.

**Table 1.** Condition properties

Condition name	Target search 1 present in trial	Target search 2 present in trial	Currently relevant item	Prospectively relevant item
Current lateralized	Yes	Yes	Left or right	Top or bottom
Prospective lateralized	Yes	Yes	Top or bottom	Left or right
Current only	Yes	No	Left or right	NA
Prospective only	No	Yes	NA	Left or right

The experiment was divided in two sessions with load (1 or 2) blocked across sessions, and order of sessions counterbalanced across participants. Each session started with an instruction followed by a practice block of 26 trials. Subsequently, participants performed 12 blocks of 20 trials each. The status of the lateralized item (current or prospective template) was randomized within these blocks. Between each block, there was a short break during which participants received feedback on their accuracy in the preceding block. During the practice blocks, participants received feedback after each trial.

##### Stimuli.

Participants were seated in a sound-attenuated and electrically shielded room and were instructed to sit as relaxed as possible to prevent EMG and electrode movement noise in the EEG signal. The chair and screen were placed at individually adjusted height, and the two response buttons on the armrests made sure the arms were in a relaxed and comfortable position. The viewing distance was 75 cm from a 22 inch video monitor (Samsung Syncmaster 2233, 1680 × 1050 pixels at 120 Hz). The stimuli were created using OpenSesame, version 2.9.0 (Mathôt et al., 2012), a Python-based graphical experiment builder.

The background color was gray (81 Cd/m^2^). The fixation cross was a black plus sign (0.6° of line length). In the memory display, the four colored circles were presented 1.7° left, right, above, and below the fixation cross. The circles had a radius of 0.6° with a black outline of 0.09° that was full (current template), dashed (prospective template), or dotted (irrelevant). The visual search display contained six colored circles, presented equidistantly on an imaginary circle with a radius of 4°. These parameter values fall well within a range of commonly used values that have been shown to produce lateralized EEG patterns, such as the CDA (Carlisle et al., 2011; Woodman and Arita, 2011; Gunseli et al., 2014). The arrowhead pointing either left or right (< or >) was drawn in the center of each circle (always three left- and three right-pointing arrows, randomly divided across all six items).

To encourage use of VWM and discourage verbalization of the colors of the memory items, we strictly controlled the colors used in the displays. In total, there were 12 colors created in DKL color space (Derrington et al., 1984) that were equidistant from each other in hue (from 12 to 324 degrees in steps of 24, skipping 108 and 156 because they were subjectively too similar) while being constant in the other two dimensions: contrast = 1 and luminance = 0 (i.e., isoluminant). This created an imaginary circle of 12 discrete colors (41.2 ± 4 Cd/m^2^, the slight deviation from isoluminance probably stems from usage of a not perfectly calibrated screen), with those opposite from each other being least similar in hue and those next to each other being most similar in hue. In the memory display the color needed for the first search display (current template) was randomly chosen, after which the color needed for the second search display (prospective template) was chosen as the opposite (i.e., least similar) color on the color circle. The other two colors in the memory display (not to be memorized) were chosen as the two colors exactly in between the currently and prospectively relevant color on the color circle, and thus opposite from each other. In the memorized color search (i.e., the search display in which participants needed to find the memorized color; Fig. 1*c*, left), the memorized color was displayed together with five other, dissimilar colors that were all relatively similar in hue to the memorized color. These five colors were randomly chosen from eight possible colors surrounding the memorized color on the color circle. In the duplicate color search (i.e., the search display in which participants needed to find the duplicate color; Fig. 1*c*, right), all colors were randomly chosen from the 12 possible colors and the circles with the same (target) color were placed next to each other.

##### Data recording and preprocessing.

EEG data were acquired at 512 Hz using a 64 electrode cap with the electrodes placed according to the extended 10–20 system (using a BioSemi ActiveTwo system; www.biosemi.com), and from both earlobes (used as reference). Additionally, vertical EOG (VEOG) was recorded from electrodes located 2 cm above and below the right eye, and horizontal EOG (HEOG) was recorded from electrodes 1 cm lateral to the external canthi. The HEOG was used in the detection of horizontal eye movement artifacts. All offline analyses were performed in MATLAB 2014a (The MathWorks).

EEG data were first rereferenced to the average of left and right earlobes after which a 0.1 Hz high-pass filter (least-squares FIR) was applied. Continuous EEG was epoched from −1 to 10 s surrounding the memory display onset. Epochs were baseline-normalized using the whole epoch as baseline, which improves independent component analysis (Groppe et al., 2009). Before cleaning, the data were visually inspected for malfunctioning electrodes, which were temporarily removed from the data: six subjects had one (*n* = 3), two (*n* = 1), or three (*n* = 2) malfunctioning electrodes. We used an adapted version of an automatic trial-rejection procedure as implemented in the Fieldtrip toolbox (Oostenveld et al., 2011) to detect EMG artifacts. We used a 110–140 Hz bandpass filter to capture muscle activity specifically, and allowed for variable *z*-score cutoffs per subject based on the within-subject variance of *z*-scores. This resulted in an average cutoff of 15.8 ± 2.2, which in turn resulted in an average rejection of 4.2% of all trials (minimum-maximum across subjects: 0.2%–10.4%). After trial rejection, we performed independent component analysis as implemented in the EEGLAB toolbox (Delorme and Makeig, 2004) on the clean electrodes only. Together with the VEOG signal, we visually inspected the independent component analysis components and identified those that captured eye-blinks or other artifacts that were clearly not brain-driven and removed these from the data. After this step, we interpolated the malfunctioning electrodes identified earlier using spherical spline interpolation as implemented in EEGLAB's eeg_interp.m function. Finally, we detected trials with large horizontal eye movements using the pop_artstep.m function in ERPLAB (Lopez-Calderon and Luck, 2014), applied on the HEOG signal, with a window length of 400 ms, step size of 10 ms, and an individualized threshold of 25.5 ± 4, and only during a time window of −50 to 900 ms surrounding memory display onset. The HEOG signal was high-pass filtered at 1 Hz to make eye-movement detection easier. Using these settings, we detected sudden sharp jumps in the HEOG surrounding the lateralized memory display onset and during start of the delay period, during which it was most important to keep fixation. This resulted in a rejection of 4.2% of all trials (minimum-maximum across subjects: 0.2%–15.0%).

Trials with an incorrect response in either one of the two search displays were excluded from further analyses, resulting in a rejection of 20% of all trials (minimum-maximum across subjects: 2.9%–26.3%). We also applied a two-step trimming on the basis of reaction time (RT): First trials with a response faster than 300 ms and slower than 5000 ms were rejected as they were unreliable. Subsequently, trials with a response of 3 SDs above or below the mean per condition and per search display were also excluded. This RT trimming led to a rejection of 3.3% of all trials (minimum-maximum across subjects: 2.3%–4.4%). All steps together (RT, noise, incorrect response, and horizontal eye movements) left on average 72% of all trials intact (mean/minimum-maximum percentage across subjects: Current Lateralized: 67.9/55.0–89.2; Prospective Lateralized: 65.6/49.2–90.8; Current Only: 80.3/60.8–92.5; Prospective Only: 74.7/54.2–93.3). These correct, artifact-free trials were divided according to the four conditions and the side of the lateralized memory item (left or right) during memory display. For the analysis of the second delay period, the epochs were time-locked to the button press of the first search response (which demarcated the onset of the second delay).

##### CDA.

**Figure 2.**
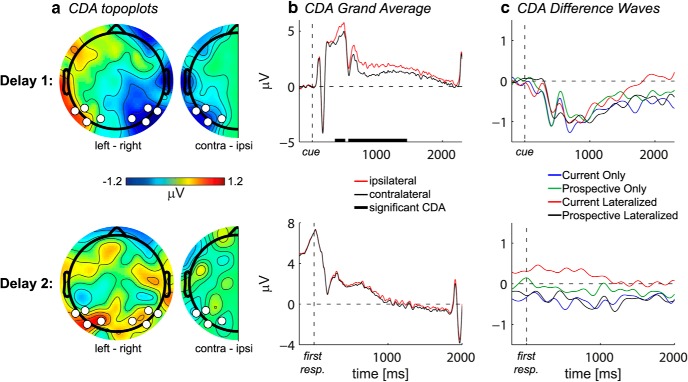
CDA. ***a***, The grand average scalp distribution of the CDA averaged over the time interval in which the CDA was significant in the first delay period. Full topographies, ERPs of trials with the lateralized memory item on the right subtracted from those with the memory item on the left. Half topographies, ERPs at ipsilateral electrodes subtracted from those at contralateral electrodes, collapsed across hemispheres. White dots indicate electrodes of which activity is plotted in ***b*** and ***c***. ***b***, The grand average ERPs at the average of P5/6, P7/8, PO3/4, and PO7/8, contralateral (black) and ipsilateral (red) to the lateralized memory item. The ERPs were low-pass filtered at 40 Hz. Thick black line on the *x*-axis indicates the time period during which the difference between the contralateral and ipsilateral ERPs was significant at *p* < 0.05 cluster-corrected. ***c***, The CDA per condition, calculated as the ERP contralateral minus ipsilateral to the lateralized memory item. The difference waves were low-pass filtered at 5 Hz for visualization purposes. First and second row are first and second delay, respectively.

To obtain ERPs, epochs were baseline-corrected using a prestimulus baseline period from −200 to 0 ms relative to the onset of the memory display, after which the signals were 40 Hz low-pass filtered. The CDA was obtained by comparing the ERP from posterior electrodes contralateral versus ipsilateral to the hemifield in which the memory item was presented (see below for electrode selection procedures). Topographically, the CDA was visualized by subtracting average delay activity after memory items presented in the right hemifield from activity after memory items presented in the left hemifield (see Fig. 2*a*).

To assess the CDA during the second delay, we time-locked it to the disappearance of the first search display (as triggered by the first response), while we used the same baseline as for the CDA during the first delay (i.e., −200 to 0 ms relative to memory display onset). Consequently, the second delay CDA is a response-locked ERP relative to a stimulus-locked baseline, with large and variable time intervals. As a control analysis, we also computed the second delay CDA with a preresponse baseline and with a whole-trial baseline, which did not produce qualitative differences (data not shown).

##### Time-frequency decomposition: Laplacian.

Before time-frequency decomposition, the surface Laplacian of the EEG data was estimated (Perrin et al., 1989), which is equivalent to the current source density approach. This method acts as a spatial high-pass filter by accentuating local effects while filtering out distant effects due to volume conduction; thus, it sharpens the EEG topography, which is especially relevant for phase-based connectivity analyses (Cohen, 2014; Kayser and Tenke, 2015). For estimating the surface Laplacian, we used a 10th-order Legendre polynomial, and λ was set at 10^−5^.

##### Morlet wavelet convolution.

The epoched EEG time series were decomposed into their time-frequency representations using custom-written MATLAB scripts. The time series were convolved with a set of Morlet wavelets with frequencies ranging from 1 to 40 Hz in 25 logarithmically spaced steps. The complex wavelets were created by multiplying sine waves (*e*^*i*2π*ft*^ where *i* is the complex operator, *f* is frequency, and *t* is time) with a Gaussian (*e*^−*t*^2^/2*s*^2^^, where *s* is the width of the Gaussian). The width of the Gaussian was set as *s* = δ/(2π*f*), where δ represents the number of cycles of each wavelet, logarithmically spaced between 3 and 12 to have a good trade-off between temporal and frequency precision. Convolution was applied in the frequency domain: the Fast Fourier Transform was applied to both the EEG data and the Morlet wavelets, and these were multiplied in the frequency domain, after which the inverse Fast Fourier Transform was applied. The frequency-specific power at each time point was defined as the squared magnitude of the complex signal resulting from the convolution: [real(*Z_t_*)^2^ + imag(*Z_t_*)^2^]. Power was averaged over trials per condition, after which the power was decibel normalized [dB Power*_tf_* = 10 × log10(Power*_tf_*/Baseline Power*_f_*)], where for each channel and frequency the baseline was defined as the condition-averaged power in the period 500 to 200 ms before onset of the memory display. Power during the second delay period was thus response-locked power relative to this prestimulus baseline.

##### Connectivity analyses: debiased weighted phase-lag index (dwPLI).

We estimated intersite phase clustering, a measure of phase-based functional connectivity between regions (Cohen, 2014), by means of the debiased weighted phase-lag index (dwPLI) (Stam et al., 2007; Vinck et al., 2011). For this, the sign of the instantaneous phase difference between channels is averaged over trials after which the absolute value is taken. If all phase difference values have the same sign (i.e., one channel is either consistently leading or lagging in phase relative to the other), the PLI will be high. If the phase difference is consistent over trials, but close to zero or π radians, PLI will be low (because the signs will cancel each other out). This measure thus ignores random as well as zero or π phase-lag, thereby controlling for spuriously inflated connectivity due to volume conduction (Srinivasan et al., 2007; Stam et al., 2007). The PLI gives high values when the phase difference has a consistence sign across trials, even when the phase difference itself is not consistent (high spread). To correct for this potential bias, we used the debiased weighted version, which weights the phase difference according to their distance from the real axis (Vinck et al., 2011). We computed the wPLI (Vinck et al., 2011, their Eq. 8) and used a further debiasing term to correct for some inflation due to sample size as implemented in Fieldtrip's ft_connectivity_wpli.m function (Cohen, 2014, their Eq. 26.7). This value was calculated for each time-frequency point.

Because the dwPLI is non-normally distributed, we applied nonparametric testing already at the within-subject level. For each time-frequency point, the phase angle trial-vector of one channel was randomly shuffled with respect to the other channel over 500 iterations. This removes any consistent relationship between channels across trials while maintaining the autocorrelative structure of each phase angle time series. At each iteration, the dwPLI was calculated on this “surrogate” data. This procedure results in distributions of dwPLI values under the null-hypothesis of no connectivity. The real connectivity values were normalized with *z*-transformation using these distributions (Maris and Oostenveld, 2007; Cohen, 2014), after which the group level statistics were applied (see Statistics).

##### Selection of electrodes, frequency bands, and time windows of interest.

All selection procedures were performed on the grand average data and were thus orthogonal to and unbiased by condition differences. Only after these selection procedures, we statistically compared the conditions of interest (see subsection “Statistics”). Based on previous studies, we first selected P5/6, P7/8, PO3/4, PO7/8, and O1/2 for the CDA analysis (McCollough et al., 2007). Visual inspection of the grand average topographical map and ERPs per channel led us to drop O1/2, thereby selecting the peak of the condition-average CDA to be most sensitive to potential condition differences. Analyses including O1/2 showed qualitatively similar results (data not shown). For the lateralized power, we initially selected the same eight electrodes as for the CDA analysis. We visually inspected the grand average time-frequency plot of the contralateral versus ipsilateral contrast and computed a topographical map of average power within the time-frequency window that showed the strongest effect (similar to Fig. 4*b*). This led us to continue time-frequency analyses with electrodes O1/2, PO3/4, and PO7/8 because P5/6 and P7/8 were too far from the peak of the effect. Analyses including these four electrodes showed qualitatively similar results (data not shown). Through group-level cluster-based permutation testing (see subsection “Statistics”), we determined the time-frequency cluster that showed a significant contralateral versus ipsilateral contrast in the grand average (see Fig. 4*a*, left, black outline).

For connectivity analyses, we initially chose as “seeds” the same six electrodes as for the lateralized α power analysis. We visually inspected the grand average topographical maps of connectivity between the three seed electrodes contralateral to the memory item and all other electrodes minus connectivity between the three seed electrodes ispilateral to the memory item and all other electrodes, creating a “lateralized connectivity index” for the whole scalp. This showed a strong lateralized connectivity effect between the seed electrodes and Pz/POz (data not shown). These two electrodes were thus selected as target electrodes for further analyses. Because lateralized power and lateralized connectivity during the second delay did not show any significant modulation as revealed by cluster-based testing, we selected a rectangular time-frequency window closely surrounding the time range of the cluster in the first delay period (i.e., 0–1000 and 0–900 ms relative to delay onset for power and connectivity, respectively; see Figs. 4*a*, second column, black outlines, 5*a*, right).

##### Statistics.

RT and accuracy for first and second search display were entered in repeated-measures ANOVAs with the within-subject factor Condition (Current Lateralized, Prospective Lateralized, Current Only, and Prospective Only). We used the Greenhouse-Geisser correction for violation of sphericity, and pairwise comparisons were corrected for multiple comparisons using the Bonferroni correction.

Statistical analyses for the EEG data were done both parametrically and nonparametrically. The first (nonparametric) step was performed on the grand average data and was thus unbiased by potential condition differences. Time-frequency maps of power (or time series for CDA) at, and connectivity between, our selected electrode groups were statistically tested for differences between electrodes contralateral and ipsilateral to the lateralized memory item using group-level permutation testing with cluster correction for multiple comparisons. For every time-frequency point (or time point for CDA), *t* values were computed for the contralateral versus ipsilateral difference and a threshold was set at a certain *p* value (≤0.05; see Results), resulting in clusters of significant time-frequency points (or time points for CDA). Next, for each time-frequency point, the sign of the power difference between contralateral and ipsilateral was randomly shuffled across subjects in 2000 iterations, and a *t* test was performed on the shuffled data in all iterations for the contralateral versus ipsilateral difference. At each permutation, the size of the largest time-frequency cluster of significant *t* values was determined, resulting in a distribution of maximal cluster sizes under the null-hypothesis of no difference between contralateral and ipsilateral. The sizes of the significant time-frequency (or time for CDA) clusters of the real data were thresholded using the percentile of the null-hypothesis distribution that corresponded to the same *p* value as for the initial *t* test (e.g., the 95th percentile for *p* < 0.05). For the nonlateralized power and connectivity analysis, a similar permutation test was used to find clusters of activity that were significantly different from zero. This nonparametric method corrects for multiple comparisons (comparing each time-frequency point) by taking into account autocorrelation in time and frequency (Maris and Oostenveld, 2007; Cohen, 2014).

As a control analysis, we also calculated power using a baseline free method for both delays, equivalent to the within-subject permutation test used for the dwPLI (see Connectivity analyses). We randomly shuffled the contralateral and ipsilateral labels over trials in each of 1000 iterations to create a distribution of raw power difference values under the null-hypothesis of no contralateral versus ipsilateral difference. The observed raw power differences were then normalized with *z*-transformation using these distributions. This control analysis produced similar results (data not shown), and we thus only report dB-corrected power.

While we used a repeated-measures ANOVA with as only factor Condition (four levels) for our behavioral analysis, our main EEG measures focused on lateralized effects and so we split the factor Condition in the EEG analysis into two factors with two levels each: Lateralized Item (current or prospective template) and Memory Load (1 or 2). Permutation testing is bivariate, thus precluding statistical inferences regarding interaction effects between these factors. We thus averaged over the time-frequency points (or time points for CDA) within significant clusters as revealed by the group-level permutation tests on the condition-average data. We then performed repeated-measures ANOVAs with the within-subject factors Lateralized Item (current or prospective template), Memory Load (1 or 2), Memory Side (left or right), and Electrode Side (left or right). If the EEG measure was nonlateralized, we excluded the factor Electrode Side. This approach can thus reveal interaction effects in time-frequency clusters based on bivariate permutation testing. All the ANOVAs mentioned above were performed using IBM SPSS Statistics, version 21.

If there was any indication that the ANOVAs on the selected time-frequency clusters would miss a possibly interesting difference between conditions (due to it being in a different time and/or frequency region), we performed the group-level cluster-based permutation test as described above, but now directly on the difference between conditions. In addition, to test whether a lateralization effect was better explained by contralateral or by ipsilateral electrodes, we performed two ANOVAs for contralateral and ipsilateral electrodes separately, thus excluding the factors Memory Side and Electrode Side. We would like to stress that these steps were based on observed data patterns and are thus exploratory; where applicable, this is indicated in Results.

## Results

### Behavior

Table 2 shows the average response time and accuracy for both search displays. On average, participants performed well (mean ± SD percentage correct: first search display: 92.2 ± 2.5; second search display: 86.6 ± 4.3), with average RTs of 876 ± 136 ms and 965 ± 176 ms for the first and second search display, respectively. Overall, participants responded faster (*t*_(19)_ = −5.5, *p* < 0.001) and more accurate (*t*_(19)_ = 9.0, *p* < 0.001) in the first than in the second search. Performance was very similar for all conditions in Search 1, except for the Prospective Only condition, where accuracy was higher (resulting in a main effect of condition, *F*_(3,57)_ = 36.1, *p* < 0.001), whereas RTs tended to be slower (*F*_(2.0,38.5)_ = 2.9, *p* = 0.069). This is the only condition where the first task involved duplicate color search rather than designated color search. Performance was also very similar for all second search conditions, except in the Current Only condition, where now participants responded both faster (*F*_(1.8,33.4)_ = 5.9, *p* = 0.008) and with higher accuracy (*F*_(3,57)_ = 93, *p* < 0.001) relative to the other conditions. Here, too, the task was duplicate color search rather than designated color search. For duplicate color search, all the relevant information was present in the search display itself. Search did not require memory for a specific color; therefore, it may have been an easier task. A separate analysis without the duplicate color search indicated overall more accurate performance for the Load 1 conditions (Current Only or Prospective Only for first or second search, respectively) than for the Load 2 conditions, across both searches (search 1: *F*_(2,38)_ = 4.4, *p* = 0.019; search 2: *F*_(2,38)_ = 23.7, *p* < 0.001), which is expected given the increased memory load.

**Table 2.** Behavioral results*^a^*

	RT (ms)		Accuracy (% correct)	
	Search display 1	Search display 2	Search display 1	Search display 2
Current only	851 ± 21	870 ± 32	92.0 ± 0.56	98.5 ± 1.00
Prospective only	925 ± 22	986 ± 25	98.0 ± 0.60	87.0 ± 0.79
Current lateralized	862 ± 19	1003 ± 25	89.2 ± 0.79	81.6 ± 0.87
Prospective lateralized	865 ± 16	1003 ± 22	89.6 ± 0.74	79.2 ± 0.88

*^a^*Values are mean ± SEM. SEM values are calculated for normalized data (i.e. corrected for between-subject variability) (Cousineau, 2005; Morey, 2008). Only correct trials are used for RT.

### CDA: first delay

As can be seen in Figure 2*b*, we observed a clear CDA in the condition-averaged time-domain EEG response during the first delay, thereby replicating many previous findings (Vogel and Machizawa, 2004; McCollough et al., 2007). Indeed, the reduced ERP amplitude at electrodes contralateral compared with ipsilateral to the memory item in the first delay period was significant during two time windows (348–506 ms and 555–1455 ms, *p* < 0.05 cluster-corrected; memory side by electrode side interaction: *F*_(1,19)_ = 30.3, *p* < 0.001) (Fig. 2*b*, top row). Importantly, there were no differences between the current and prospective template in terms of CDA (*F*_(1,19)_ = 0.001, *p* = 0.976) (Fig. 2*c*, top row), suggesting that both current and prospective items were similarly stored, regardless of their status. To verify VWM storage for both types of representation, we performed two additional ANOVAs: one for conditions with a lateralized current template (collapsed over Current Only and Current Lateralized) and one for conditions with a lateralized prospective template (collapsed over Prospective Only and Prospective Lateralized). Indeed, both currently and prospectively relevant items elicited a CDA (*F*_(1,19)_ = 15.8, *p* = 0.001 and *F*_(1,19)_ = 31.7, *p* < 0.001, respectively), suggesting VWM storage for both representations. There was also no effect of memory load (i.e., one vs two items in memory, *F*_(1,19)_ = 0.021, *p* = 0.887). This may seem at odds with previous evidence showing that the CDA increases with increasing VWM load (Luria et al., 2016). However, we point out that in our experiment one of the items was always presented on the central meridian, and hence no additional load effect was expected here on the lateralized CDA.

### Local α power: first delay

**Figure 3.**
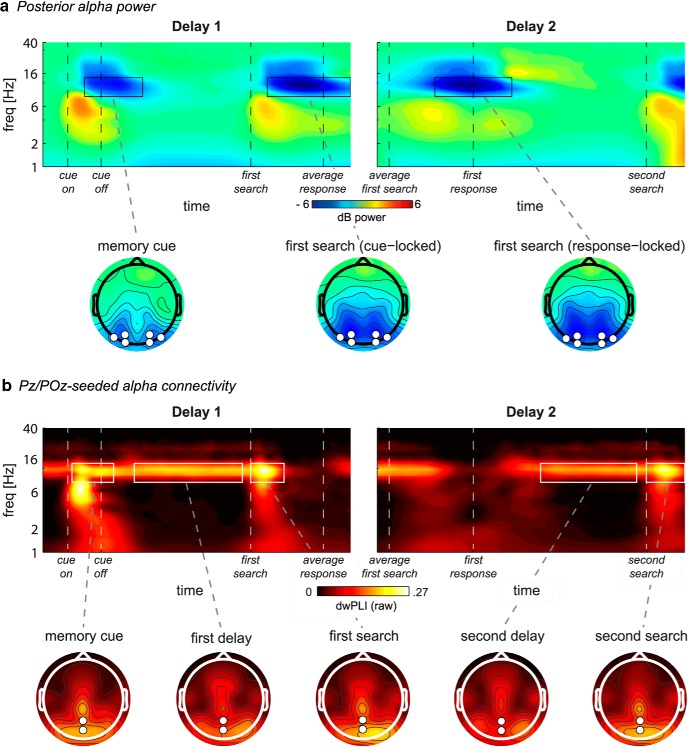
Overall task-related power and connectivity. ***a***, Top, Grand average time-frequency plots of power at the average of O1/2, PO3/4, and PO7/8, for first (left) and second (right) delay. Bottom, Grand average scalp distributions of α power (8–14 Hz) averaged over the time-frequency windows highlighted by black outlines in the time-frequency plots. Each task display is followed by overall posterior α suppression relative to baseline. ***b***, Top, Grand average time-frequency plots of connectivity (raw dwPLI) between the six lateral posterior electrodes O1/2, PO3/4, and PO7/8 and the parietal Pz/POz electrodes, for first (left) and second (right) delay. Bottom, Grand average scalp distributions of α connectivity (8–14 Hz) averaged over the time-frequency windows highlighted by white outlines in the time-frequency plots. Connectivity in the topographical maps is calculated between each electrode and Pz/POz combined. ***a***, ***b***, First delay is locked to memory display onset. Second delay is locked to first response, which coincides with second delay onset.

**Figure 4.**
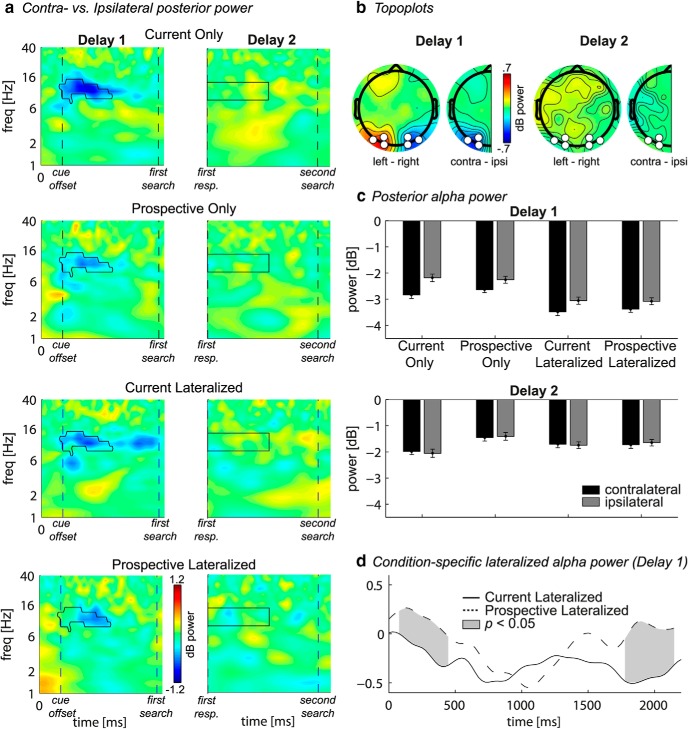
Lateralized posterior α power. ***a***, Time-frequency plots per condition (rows) of contralateral minus ipsilateral power at the average of O1/2, PO3/4, and PO7/8 for the first and second delay (columns). Black outline indicates significant power difference between contralateral and ipsilateral in grand average at *p* < 0.01, cluster corrected. For the first delay, *t* = 0, *t* = 400, and *t* = 2200 indicate the memory display onset, start of first delay, and end of first delay, respectively. For the second delay, *t* = 0 and *t* = 1800 indicate the start and end of second delay, respectively. ***b***, Full topographies, Grand average scalp distributions of α power on trials with the lateralized memory item on the right subtracted from those with the memory item on the left. Half topographies, Grand average scalp distributions of α power at ipsilateral electrodes subtracted from those at contralateral electrodes, collapsed across hemispheres. Power was averaged over the significant time-frequency cluster (first delay) or averaged over a window of 0–1000 ms by 8–14 Hz (second delay) (see ***a***, black outline). ***c***, Bars per condition of contralateral (black) and ipsilateral (gray) power averaged over the time-frequency cluster highlighted in ***a***. Error bars indicate SEM for normalized data. ***d***, Time series of contralateral minus ipsilateral α power during the first delay for the conditions where both the currently relevant and prospectively relevant item had to be remembered. Gray areas between the curves represent time points with a significant difference between the conditions after cluster correction at *p* < 0.05.

In the time-frequency domain, overall power in the α frequency band (8–14 Hz) over posterior electrodes was suppressed relative to baseline in response to visual stimulation (i.e., the memory display and both search displays; Fig. 3*a*). In addition, the α band also revealed a lateralized effect that was topographically similar to the CDA but consisted of a stronger reduction in power relative to baseline for contralateral compared with ipsilateral electrodes (Fig. 4*a*,*b*). A cluster-based permutation test confirmed this effect to be significant ∼300–1300 ms (*p* < 0.01; memory side by electrode side interaction: *F*_(1,19)_ = 30.3, *p* < 0.001). Earlier studies have found lateralized posterior α suppression during VWM maintenance and related this to mechanisms of spatially selective attention (Sauseng et al., 2005b; Thut et al., 2006; Medendorp et al., 2007; Myers et al., 2015). Importantly, lateralized α suppression was stronger for the current compared with prospective template (*F*_(1,19)_ = 5.5, *p* = 0.03), suggesting that this selective attention mechanism is, at least in part, sensitive to the status of VWM representations. Two additional ANOVAs separating contralateral and ipsilateral electrodes revealed that the sensitivity to VWM status was driven by stronger contralateral suppression (*F*_(1,19)_ = 7.1, *p* = 0.015), rather than weaker ipsilateral suppression (*F*_(1,19)_ = 0.4, *p* = 0.53) for the current template. Thus, α dissociated from the CDA in its sensitivity to VWM status. Finally, overall α suppression did reveal a nonlateralized load effect: In both ipsilateral and contralateral electrodes, it was stronger when participants had to remember two items (main effect of load: *F*_(1,19)_ = 12.6, *p* = 0.002). Previous work has suggested that bilateral α suppression reflects VWM storage, as it correlates with spatially global memory load (Fukuda et al., 2015, 2016). This finding thus also indicates that both current and prospective representations were stored in VWM in our task, in accordance with the CDA results.

In sum, we found that both lateralized α suppression and the CDA are present during late encoding and VWM maintenance, but that only lateralized α suppression is sensitive to the status of VWM representations, thus suggesting a dissociation between CDA and modulations in α power.

### Additional exploratory analyses on α power

We next performed additional analyses that were based on observed condition differences. These analyses are thus exploratory in nature, and the results are meant to complement the above findings. In the condition-specific time-frequency maps (Fig. 4*a*), we observed stronger lateralized α suppression in the Current Lateralized compared with the Prospective Lateralized condition both at the beginning and toward the end of the first delay period. Because this effect fell outside of the time-frequency cluster that we used to test for condition differences, we here averaged lateralized power over the α band, and directly tested for differences between Current Lateralized and Prospective Lateralized using cluster correction over time. Indeed, lateralized α suppression was stronger in the Current Lateralized condition during encoding (75–450 ms) and at the end of the first delay period (1775–2150 ms) (*p* < 0.05; Fig. 4*d*). Remarkably, this effect disappeared in the middle of the first delay period. This dissociation in lateralized α dynamics indicates that oscillatory activity is sustained throughout the delay period for the item relevant for the upcoming search, whereas it is not for prospectively relevant items.

### Interregional connectivity: first delay

**Figure 5.**
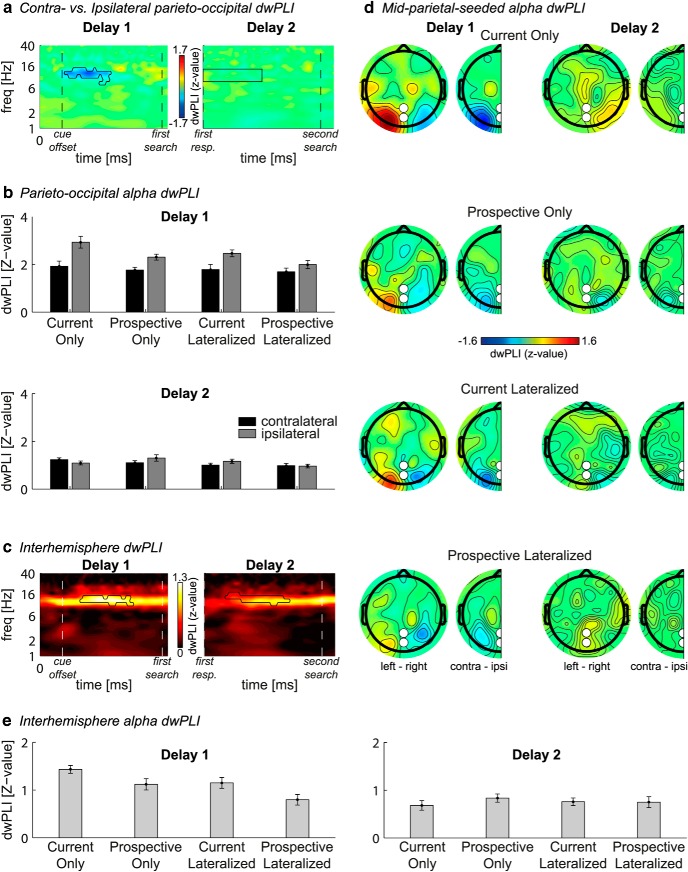
Lateralized functional connectivity. Connectivity is calculated as *z*-transformed dwPLI. ***a***, Grand average time-frequency plots of contralateral minus ipsilateral connectivity between the three lateral posterior electrodes O1/2, PO3/4, and PO7/8 and the parietal Pz/POz electrodes. Black outline indicates a significant difference between contralateral versus ipsilateral connectivity in grand average after a permutation test with cluster correction at *p* < 0.05. ***b***, Bars represent, per condition, the average contralateral (black) and ipsilateral (gray) connectivity between POz/Pz and O1/2, PO3/4, and PO7/8 within the time-frequency cluster/window highlighted in ***a***. ***d***, Scalp distributions, per condition (rows), of connectivity averaged over the significant time-frequency cluster depicted in ***a*** (first delay, first column) or averaged over a window of 0–900 ms by 8–14 Hz (second delay, second column). Topographical maps, Connectivity between each electrode and Pz/POz combined, for trials where the memory item was presented on the left minus those where it was presented on the right (full topographies), or for ipsilateral electrodes subtracted from contralateral electrodes and collapsed over hemispheres (half topographies). ***c***, Grand average time-frequency plots of connectivity between left (O1, PO3, and PO7) and right (O2, PO4, and PO8) hemisphere. Black outline indicates significant connectivity after permutation test with cluster correction at *p* < 0.001. For the first delay, *t* = 0, *t* = 400, and *t* = 2200 indicate the memory display onset, start of first delay, and end of first delay, respectively. For the second delay, *t* = 0 and *t* = 1800 indicate the start and end of second delay, respectively. ***e***, Bars represent average connectivity within the significant time-frequency cluster, per condition. Error bars indicate SEM for normalized data.

In addition to local α power, we also analyzed interregional functional connectivity. Pz/POz-seeded α connectivity with posterior electrodes was elevated in response to visual stimulation (i.e., the memory display and both search displays) and continued into both delay periods (Fig. 3*b*). In addition, connectivity showed a lateralized effect that was remarkably similar to local lateralized α, both in terms of the frequency band (8–14 Hz) and timing (during the first delay period) (compare Fig. 4*a* with Fig. 5*a*). That is, during maintenance, reduced contralateral α power was accompanied by reduced α phase synchronization between these contralateral electrodes and two mid-parietal electrodes (Pz/POz; 400–1300 ms, *p* < 0.05, cluster-corrected; memory side by electrode side interaction: *F*_(1,19)_ = 16.4, *p* = 0.001; Fig. 5*a*, black outline). Importantly, similar to the lateralization effect in local α suppression, this lateralized connectivity effect was stronger when the lateralized memory item was the current compared with prospective template (*F*_(1,19)_ = 5.7, *p* = 0.027). Two additional ANOVAs separating contralateral and ipsilateral electrodes revealed that the sensitivity of lateralized connectivity to VWM status was driven by stronger ipsilateral connectivity (*F*_(1,19)_ = 14.4, *p* = 0.001), rather than weaker contralateral connectivity (*F*_(1,19)_ = 1.1, *p* = 0.31) for the current template. Surprisingly, this is in contrast to the lateralized α power effect, which was driven by contralateral electrodes. To investigate the relationship between hemispheres more directly, we calculated interhemispheric connectivity between left and right lateral posterior electrodes (Fig. 5*c*,*e*). Interhemispheric α band connectivity was significant at *p* < 0.05 during the whole trial, so we set the threshold for the permutation test at a conservative *p* < 0.001 to obtain the peak of the effect (Fig. 5*c*, black outline). Supporting the other results in the time-frequency domain, interhemispheric connectivity was stronger when the lateralized memory item was the current compared with prospective template (*F*_(1,19)_ = 13.3, *p* = 0.002). Together, these connectivity results suggest that a parieto-occipital network is involved in selective internal attention, and that the mid-parietal Pz/POz electrodes reflect a hub in this network. Moreover, this network seems sensitive to the status of the representation in visual working memory.

### Second delay analyses

During the second delay period, there was no CDA present (Fig. 2*b*, bottom row), even though the item was still remembered (as indicated by behavioral performance). Similarly, there was no lateralized modulation of α suppression (Fig. 4*a*, right column), or of connectivity (Fig. 5*a*, right). We found clear interhemispheric posterior α connectivity during the second delay, but there were no effects of status of the lateralized memory item (Fig. 5*c*,*e*, right; *F*_(1,19)_ = 0.9, *p* = 0.364). The absence of such lateralized memory markers is of interest, given that in the Prospective Lateralized and Prospective Only condition, the item that was needed for the second search display was initially presented lateralized.

**Figure 6.**
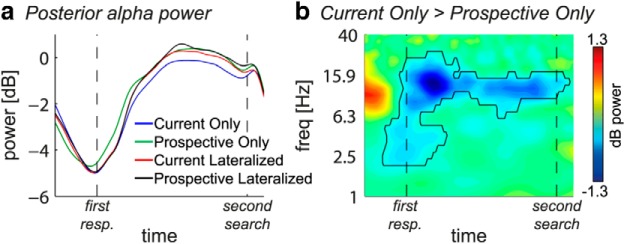
Nonlateralized posterior α power in second delay. ***a***, Time series of α power per condition averaged over O1/2, PO3/4, and PO7/8 during the second delay, independent of lateralized memory item location. ***b***, Time-frequency plot of condition difference (Current Only − Prospective Only) during the second delay. Black outline indicates significant condition difference at *p* < 0.05 cluster corrected. In the time-frequency plot, *t* = 0 and *t* = 1800 indicate the start and end of second delay, respectively.

Additional exploratory analyses suggested α suppression in both contralateral and ipsilateral electrodes to be stronger in the Current Only compared with all other conditions (see Fig. 4*c*, delay 2). We therefore analyzed overall nonlateralized α power averaged over all six posterior electrodes and independent of location of the memory item (Fig. 6). The time series (Fig. 6*a*) show a strong overall α suppression in response to the first search display (see also Fig. 3*a*), followed by an increase in α power back to baseline levels. This relative α enhancement back to baseline was significantly weaker in the Current Only compared with the Prospective Only condition during the whole second delay period (−175 to 1950 ms, *p* < 0.05 cluster-corrected) (Fig. 6*b*). In the Current Only condition, no prospective template needed to be retrieved after finishing the first search. In contrast, in the Prospective Only condition, retrieval of the prospective template was needed after the first search. Thus, this pattern of findings suggests that α enhancement may play a functional role during retrieval of representations that were thus far not relevant.

## Discussion

We recorded EEG during the maintenance of targets that were required for either the first or the second of two consecutive search tasks, to investigate the dissociation between current and prospective relevance of VWM representations for visual search. The most important result was stronger hemisphere-specific modulation of posterior α power during the encoding and maintenance of currently relevant compared with prospectively relevant representations. Accompanying this pattern, modulations in α phase synchronization between mid-parietal and hemisphere-specific parieto-occipital cortex were stronger for current templates. In contrast, the CDA did not show any differences and was equally present for both current and prospective templates, indicating that both types were stored (a conclusion corroborated by the load sensitivity of nonlateralized α power). Together, our findings distinguish between storage and priority in working memory, and provide novel evidence that posterior α-band oscillatory dynamics, both in terms of local power modulations as well as interregional connectivity, are sensitive to the functional role of current and future goals in visual search, whereas the CDA is not.

### Current relevance in VWM

Our α power results are consistent with studies showing that prioritization based on anticipated target location (Sauseng et al., 2005b; Thut et al., 2006; Spaak et al., 2015), anticipated saccade direction (Medendorp et al., 2007), or memory location in a memory display (van Dijk et al., 2010; Zumer et al., 2014; Myers et al., 2015), leads to stronger posterior α suppression in regions contralateral to the relevant hemifield. This lateralization is thought to reflect selection of task-relevant, and suppression of task-irrelevant information (Jensen and Mazaheri, 2010; Romei et al., 2010; Zumer et al., 2014). We extend this by showing that lateralized α power not only reflects the selection and rehearsal of relevant information but is sensitive to differences in priority between different VWM representations for search.

Prominent models describe VWM as an emergent property of attentional mechanisms interacting with early visual areas (Awh and Jonides, 2001; Postle, 2006; Olivers, 2008; Chun, 2011; Kiyonaga and Egner, 2013), which is supported by empirical data revealing shared neural mechanisms (e.g., Pasternak and Greenlee, 2005; Gazzaley and Nobre, 2012). One caveat in our design is that the lateralized α modulations we observed could reflect visual spatial attention directed toward the relevant targets. However, given the aforementioned proposed overlap in mechanisms between attention and VWM, it becomes difficult to distinguish between the two. Moreover, other studies found similar lateralized α modulations after retro-cues presented during maintenance, directing attention within VWM (Myers et al., 2015; Schneider et al., 2015, 2016), indicating that lateralized α modulations do not specifically reflect external attention. Finally, the time course of the lateralized α modulation reported here suggests dynamic changes in priority within VWM: Current relevance-related α lateralization showed a transient drop in the middle of the maintenance period, after which it reemerged well into the delay period, before the first search display (Fig. 4*d*). Interestingly, prospective templates showed the reverse: Lateralization only appeared in the middle of the maintenance period and disappeared again before the first search display. This opposite effect suggests a switch in the processing of representations with differential priority, within VWM.

Some evidence suggests that it is ipsilateral α enhancement, rather than contralateral suppression, that reflects the allocation of visuospatial attention, through the inhibition of task-irrelevant information (Kelly et al., 2006; Rihs et al., 2007; Sauseng et al., 2009; Händel et al., 2011). Although our results (specifically stronger parieto-occipital connectivity with ipsilateral electrodes) do not exclude a functional role for ipsilateral α enhancement, our power results convincingly show that contralateral α suppression specifically is sensitive to VWM status, thus supporting a role for contralateral α suppression in active processing (Ikkai et al., 2016).

The special status of the current template was further corroborated by differences in connectivity. Long-range phase-coupling of α oscillations between regions may underlie top-down modulation of local processing during maintenance, reflecting a possible mechanism for selection of task-relevance within VWM (Sauseng et al., 2005a; Capotosto et al., 2009; Palva and Palva, 2011). In support of this, we observed such functional connectivity to be modulated by VWM status, as current relevance elicited stronger α phase synchronization between a mid-parietal region and parieto-occipital regions ipsilateral to the hemifield in which the memory was presented, and between left- and right-hemisphere parieto-occipital regions. This implicates the involvement of a parieto-occipital network with a mid-parietal hub in VWM prioritization, in line with the involvement of posterior parietal cortex in top-down control (Silver and Kastner, 2009; Shomstein, 2012).

Although the prefrontal cortex also has been implicated in top-down control during both attention (Bressler et al., 2008; Capotosto et al., 2009) and VWM (Sauseng et al., 2005a; Gazzaley and Nobre, 2012; Kuo et al., 2016), we did not observe frontoparietal or fronto-occipital α-band connectivity that dissociated current from prospective relevance. Possibly, oscillatory γ activity or cross-frequency α-γ coupling within or between regions is sensitive to this subtle dissociation (Palva and Palva, 2007; Jensen and Mazaheri, 2010; Van Vugt et al., 2014). However, γ activity is difficult to reliably estimate with scalp EEG (Muthukumaraswamy, 2013).

Finally, our results indicate that the CDA is not sensitive to the current relevance of the VWM representation. This corroborates earlier findings that the CDA is also insensitive to the type of task for which the memorandum has to be used (Gunseli et al., 2014). It also means that the relationship between CDA and lateralized α suppression is less clear than previously claimed (van Dijk et al., 2010). Indeed, some evidence suggests a dissociation between the two measures (Fukuda et al., 2015, 2016). Importantly, with respect to visual search, our results show that, although search templates may trigger a CDA, the presence of a CDA as such is not diagnostic for the representation of a search template (Woodman and Arita, 2011; vis-à-vis Carlisle and Woodman, 2011; Woodman et al., 2013).

### Prospective relevance in VWM

We found that prospective relevance elicited less α lateralization than current relevance, potentially reflecting a qualitative change in neural representation. Interestingly, whereas previous lines of evidence suggest that prospective representations are stored in an “activity-silent” state, for instance through changed synaptic weights (Mongillo et al., 2008) or short-term potentiation (Erickson et al., 2010), as potentially stored in prefrontal cortex (Stokes, 2015), our CDA results, as well as the load sensitivity of overall α power, suggest instead an activity-based storage mechanism also for prospective items. Several factors may play a role. First, although previous studies combined two categorically distinct and rather easy tasks from different cognitive domains (e.g., remember a word and a simple visual pattern), our task required participants to maintain two items from the same (visual) domain, which were moreover not easy to distinguish from distractors in the search displays. Easy categorical distinctions may allow for long term memory to be invoked without information loss, resulting in silent working memory, whereas our tasks demand active maintenance to prevent loss of information. Second, studies have used different measures: Whereas earlier studies used task or category decoding accuracy, we used the CDA's lateralization as a measure for storage. It may be that, although the actual content of the memory is not longer represented through activity, the index, as would be reflected by the CDA, still is acting as a pointer for retrieval. Alternatively, the CDA may reflect not active firing but the subthreshold modulation of membrane potentials until reactivation (Mendoza-Halliday et al., 2014; Wolff et al., 2015), which would indeed be consistent with an “activity-silent” state.

### Second delay period

The second delay period did not show any lateralization effects, precluding strong inferences on the changing status of VWM representations after the first search. It may be that the storage and prioritization of VWM representations are still accomplished by active neural mechanisms but are no longer retinotopically specific during the second delay, thus losing its lateralized signature. Interestingly, we observed stronger overall α enhancement for conditions where the item relevant for the second search was retrieved, suggesting that α enhancement plays a functional role during selective retrieval of representations from VWM that were thus far irrelevant (Waldhauser et al., 2012). Alternatively, it may reflect dropping the first template, without replacing it with the second template. However, it is important to mention some analysis-specific difficulties with this time interval. It always directly followed the first search and response, possibly contaminating subsequent EEG signals. Moreover, the second delay occurred quite late after our baseline time window, potentially reducing the signal-to-noise ratio. Future studies should more directly test the hypothesis of a switch in priority between the two search tasks.

### Conclusion

In conclusion, we have shown that lateralized α network dynamics are sensitive to the dissociation between current and future relevance in VWM-driven visual search. Although currently and prospectively relevant representations are both stored in VWM, as they both elicited lateralized slow evoked potentials (CDA), the currently relevant representation has a prioritized state, allowing it to directly interact with visual attention. Posterior α-band oscillation appears to be the mechanism by which the working memory system accomplishes such prioritization.
